# Developing a simple and rapid method for cell-specific transcriptome analysis through laser microdissection: insights from citrus rind with broader implications

**DOI:** 10.1186/s13007-024-01242-y

**Published:** 2024-07-27

**Authors:** Xuehan Mei, Kaijie Zhu, Danni Yan, Huihui Jia, Wangyao Luo, Junli Ye, Xiuxin Deng

**Affiliations:** National Key Lab for Germplasm Innovation & Utilization of Horticultural Crops, Huazhong Agricultural University, Wuhan, 430070 Hubei China

**Keywords:** Laser microdissection, Single-cell sequencing, RNA sequencing, Citrus rind, Cell isolation, Cell-specific, Transcriptome

## Abstract

**Background:**

With the rapid development of single-cell sequencing technology, histological studies are no longer limited to conventional homogenized tissues. Laser microdissection enables the accurate isolation of specific tissues or cells, and when combined with next-generation sequencing, it can reveal important biological processes at the cellular level. However, traditional laser microdissection techniques have often been complicated and time-consuming, and the quality of the RNA extracted from the collected samples has been inconsistent, limiting follow-up studies. Therefore, an improved, simple, and efficient laser microdissection method is urgently needed.

**Results:**

We omitted the sample fixation and cryoprotectant addition steps. Instead, fresh samples were embedded in Optimal Cutting Temperature medium within 1.5 ml centrifuge tube caps, rapidly frozen with liquid nitrogen, and immediately subjected to cryosectioning. A series of section thicknesses of citrus rind were tested for RNA extraction, which showed that 18 μm thickness yielded the highest quality RNA. By shortening the dehydration time to one minute per ethanol gradient and omitting the tissue clearing step, the resulting efficient dehydration and preserved morphology ensured high-quality RNA extraction. We also propose a set of laser microdissection parameters by adjusting the laser power to optimal values, reducing the aperture size, and lowering the pulse frequency. Both the epidermal and subepidermal cells from the citrus rind were collected, and RNA extraction was completed within nine hours. Using this efficient method, the transcriptome sequencing of the isolated tissues generated high-quality data with average Q30 values and mapping rates exceeding 91%. Moreover, the transcriptome analysis revealed significant differences between the cell layers, further confirming the effectiveness of our isolation approach.

**Conclusions:**

We developed a simple and rapid laser microdissection method and demonstrated its effectiveness through a study based on citrus rind, from which we generated high-quality transcriptomic data. This fast and efficient method of cell isolation, combined with transcriptome sequencing not only contributes to precise histological studies at the cellular level in citrus but also provides a promising approach for cell-specific transcriptome analysis in a broader range of other plant tissues.

**Supplementary Information:**

The online version contains supplementary material available at 10.1186/s13007-024-01242-y.

## Introduction

With the rapid development of single-cell sequencing technology, histologic studies have progressed to examine cellular level phenomena [1–3]. Techniques such as microcapillary aspiration and protoplast extraction have been employed to isolate specific cell types. However, microcapillary aspiration often yields poor RNA quality, which poses challenges for downstream analysis, while protoplast extraction techniques prove to be complicated and time-consuming [4–8]. These challenges have led to the exploration of alternative methods for cell isolation, one of which is laser microdissection (LMD).

LMD utilizes a high-resolution microscopy-assisted laser beam system to precisely excise cell areas from Polyethylene Naphthalate (PEN) membrane slides, with cells being collected in a sample tube by gravitational force or laser pulse ejection [9–11]. The isolated cells can then be utilized for downstream omics analysis, including transcriptome, proteome, metabolome, and epigenetic modification analyses [12–15]. Consequently, the LMD technique serves as a valuable tool for isolating specific cells or tissues to understand functional differentiation and global gene profiling, including the identification of low-abundance, tissue-specific, or cell-specific mRNAs. It also reveals spatial expression patterns of target genes. Its application in the shoot apical meristem cells of lettuce led to the identification of the *LsSOC1* gene, which encodes a protein that plays a role in promoting early bolting under extreme temperature environments [16]. To date, the LMD technique has been employed in a wide range of plant species and tissues, such as in tomato peel, apple peel, cucumber phloem, strawberry flower tissue, Arabidopsis shoot apical meristem, and rice root [17–22].

However, traditional LMD methods have some limitations. First, the operational procedures are complicated and time-consuming, requiring a tedious process of sample preparation and skilled personnel to successfully isolate the desired tissue areas. This makes the technique inaccessible to the majority of researchers. Second, extracting high-quality RNA from the collected samples for follow-up studies is challenging. Therefore, there is a need for a simpler and more efficient LMD method that researchers can reference to enable greater utilization of this technology. In an earlier study, LMD was utilized to separate citrus rind for microarray analysis, revealing functional differentiation in epidermal and subepidermal cells [23]. Unfortunately, the technical details were incomplete, and the sensitivity and dynamic range of microarray analysis were limited. RNA Sequencing (RNA-Seq), a technique for studying gene expression by directly sequencing RNA molecules, offers higher sensitivity and a broader dynamic range, as it can enable the detection of low-abundance transcripts, discovery of new transcripts and variants, and make precise quantitative analysis [24].

In this study, we established a rapid and efficient suite of experimental protocols for citrus rind by proposing a set of the LMD parameters and protocols, providing a reference for accurate histological studies of other tissues. Our method combines rapid cryosectioning with LMD, significantly reducing the time needed for the experiment. High-quality RNA can be obtained in just nine hours, and this method was successfully applied to downstream RNA-Seq analysis, revealing significant differences in gene expression patterns between the epidermal and subepidermal cells in citrus rind. The method we have developed not only advances the possibilities for detailed histological studies in citrus, but also lays the groundwork for the adaptation of this approach to a wider variety of plant tissues, thereby expanding the potential for cell-specific transcriptome analysis across diverse plant species.

## Results

### Direct embedding of fresh samples for cryosectioning

**Fig. 1:**
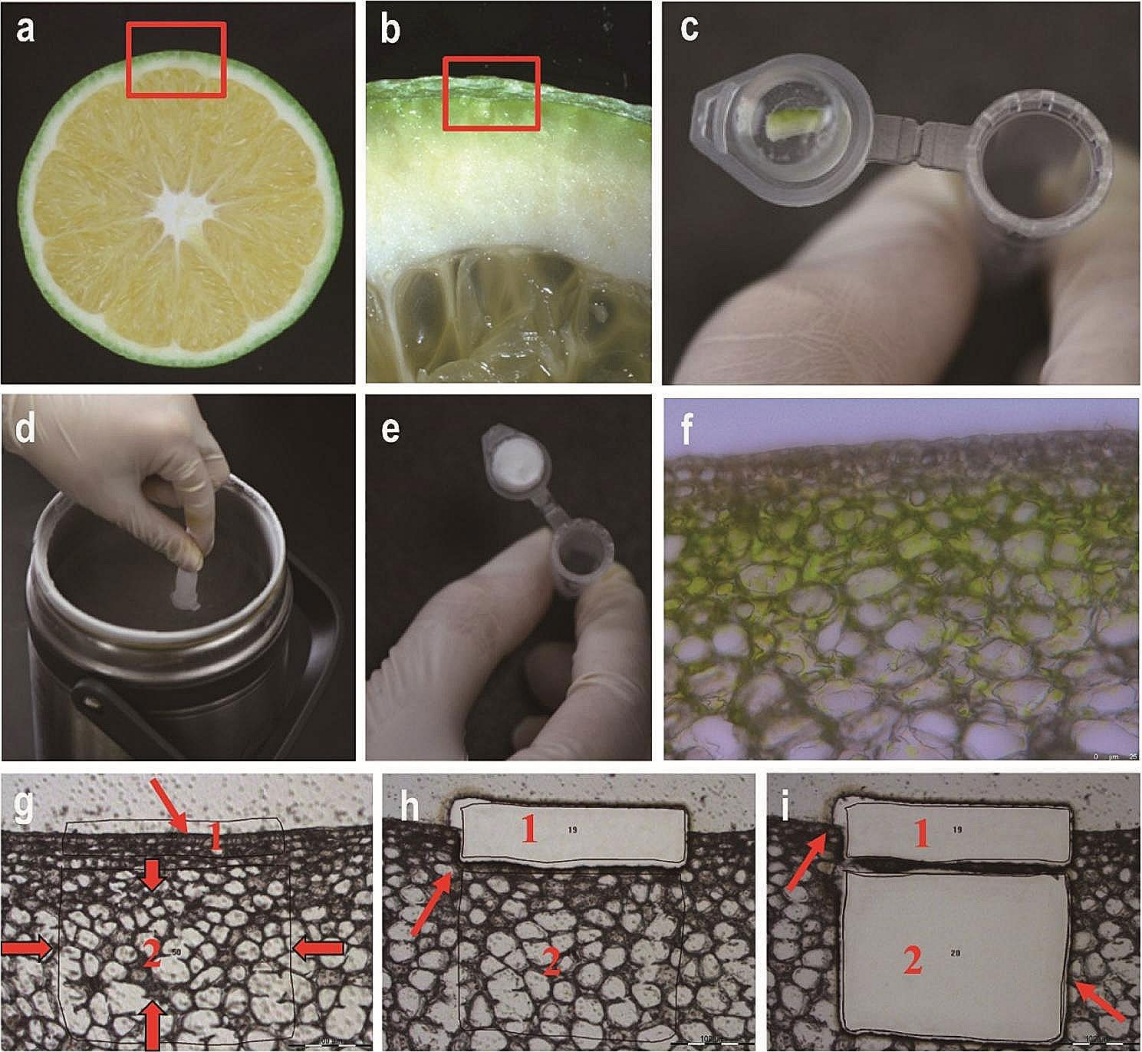
Simple workflow for LMD of citrus rind. The workflow proceeds in the order shown in the diagram. The red boxes show the sampling site. Red arrows mark the cutting regions. 1: Epidermal cells. 2: Subepidermal cells. Images were captured under a 20x magnification microscope. (**a**) Cross-section of a citrus fruit. (**b**) Enlarged view of the fruit cross-section. (**c**) The sample was embedded in the cap of a 1.5 ml centrifuge tube using OCT medium as the embedding agent. (**d**) The sample was rapidly frozen in liquid nitrogen. (**e**) Post-freezing in liquid nitrogen. (**f**) Cryosectioning to produce slices. (**g**) Prior to LMD. (**h**) Isolation of epidermal cells. (**i**) Isolation of subepidermal cells

Currently, LMD primarily relies on two sample sources: paraffin sectioning and cryosectioning. While paraffin sectioning is suitable for tissues with high water content, large vacuoles, or voids, it is a complicated and time-consuming process. Conversely, cryosectioning is simpler and faster, but it can lead to ice crystal formation when plant tissues are directly frozen, resulting in cell rupture and potential impacts on morphology, structure, and subsequent experiments. Therefore, tissues are typically fixed and cryoprotected prior to cryosectioning, which adds complexity to the process. In this study, we adopted a simplified approach. The citrus rind was cut into pieces measuring 8 mm x 1 mm x 1 mm and directly embedded in the lid of a 1.5 ml centrifuge tube filled with Optimal Cutting Temperature (OCT) medium (Fig. 1a, b, c). The samples were then immediately frozen in liquid nitrogen for cryosectioning (Fig. 1d, e). The resulting sections demonstrated well-preserved cell morphology, high integrity, and were suitable for downstream experiments (Fig. 1f). By omitting the fixation and cryoprotection steps, we were able to complete the process within minutes, without the need for specialized embedding molds or other consumables. This efficient workflow may facilitate rapid cryosectioning of other plant samples (Fig. 1).

### Identifying appropriate section thickness is essential for high-quality RNA extraction

**Fig. 2:**
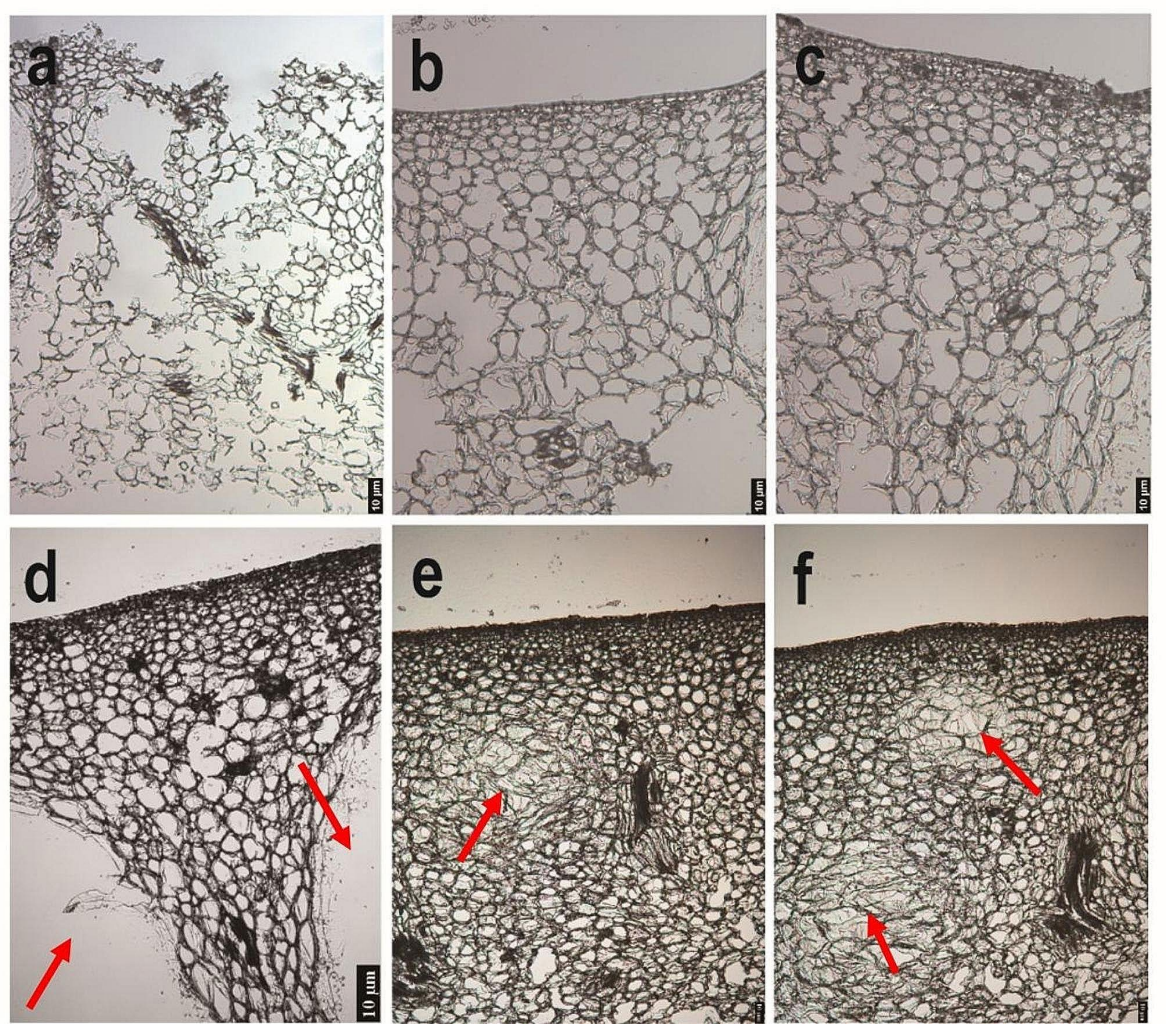
Light microscopy images showcase the morphology of the citrus rind at varying slice thicknesses. Images **a**–**d** correspond to tissue thicknesses of 8 μm, 10 μm, 12 μm, and 14 μm, respectively. Images **e** and **f** both correspond to a tissue thickness of 18 μm. Red arrows point to oil glands, which are specialized structures in the citrus rind that primarily store and secrete essential oils. All images were obtained using a 20x magnification microscope

**Fig. 3:**
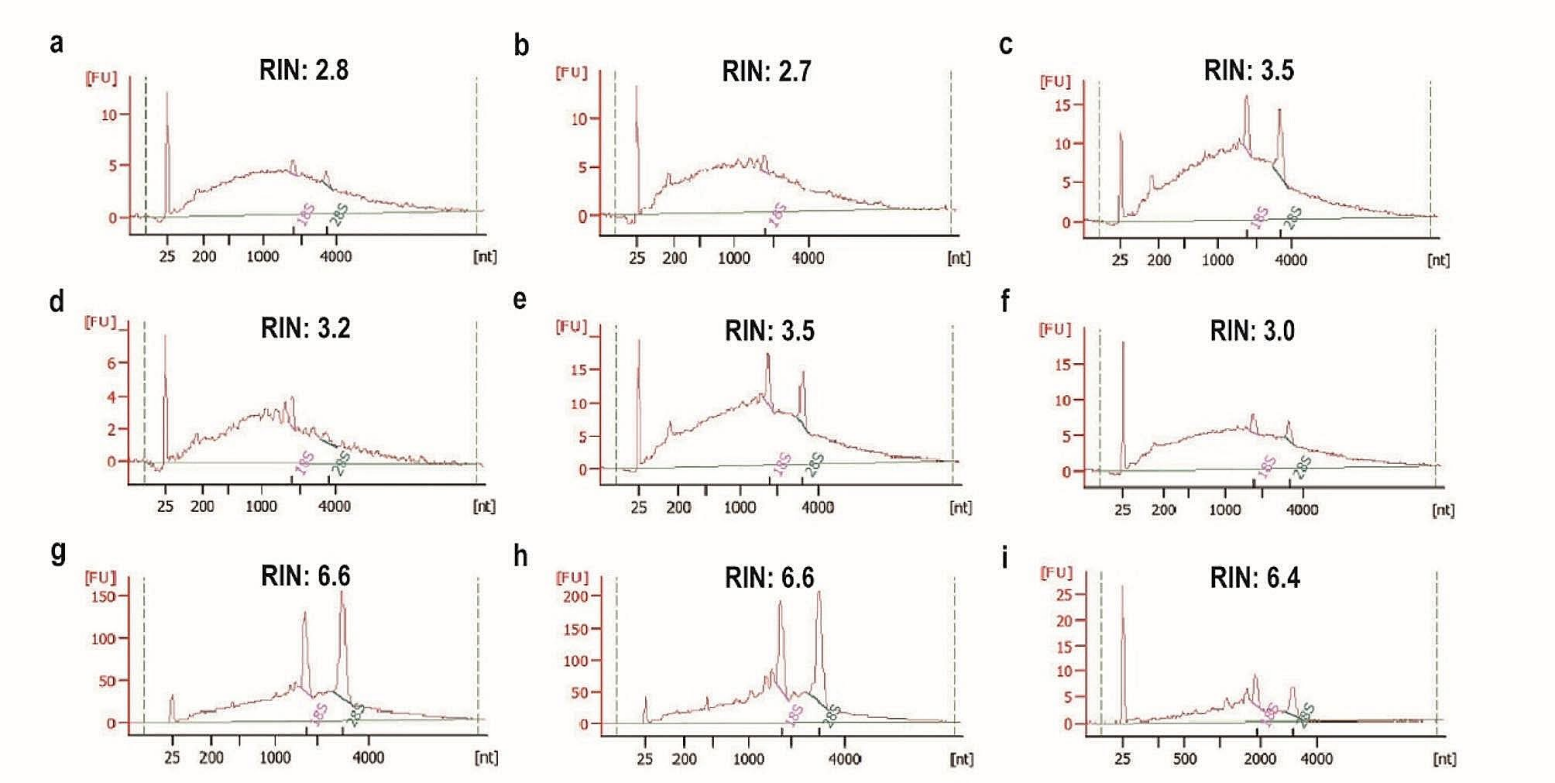
Analysis of RNA quality from LMD of citrus rind cryosections at varying thicknesses. Sections of 10 μm (**a**, **b**), 12 μm (**c**, **d**), 14 μm (**e**, **f**), and 18 μm (**g**, **h**, **i**) are included. The cells represented in plots a, b, e, g, and h are from the epidermal layer, while plots c, d, f, and i represent cells from the subepidermal layer

We investigated the impact of section thickness on RNA integrity. Most previous studies applying the LMD technique have used 8–10 μm sections, so we initially tested an 8 μm thickness. However, the results revealed significant cellular fragmentation, which complicated the cryosectioning process (Fig. 2a). When we increased the section thickness to 10 μm, cell fragmentation was reduced, but the quality of the extracted RNA remained poor; the RNA integrity numbers (RINs) were a mere 2.8 and 2.7 (Figs. 2b and 3a and b). Further attempts to optimize the process with 12 μm and 14 μm sections resulted in decreased cellular damage but did not significantly improve RNA quality (Figs. 2c and d and 3c, d, e and f). Upon careful inspection of the rind cell sizes, we found that most cells had diameters ranging from approximately 7 μm to 18 μm. Consequently, we adjusted the section thickness to 18 μm to ensure that each section would contain at least one layer of intact cells. Repeated experiments confirmed that an 18 μm section thickness not only maintained satisfactory cellular morphology but also guaranteed the quality of the RNA, resulting in an RIN of 6.6 (Figs. 2e and f and 3g, h and i). Overall, we suggest that the thickness of cryosections should match the cell diameter to ensure that each section contains at least one layer of intact cells, maximizing the quality and integrity of RNA.

### Simplifying dehydration conditions and omitting clearing steps to improve the efficiency and manageability of LMD

Due to the high water content of plant cells, dehydration is a necessary step prior to conducting LMD. However, the balance is delicate: excessive dehydration comes with a risk of RNA degradation, while insufficient dehydration hampers precise excision. Moreover, the clearing step that follows dehydration enhances tissue visualization, but the organic solvents involved can negatively affect cells and extend the experimental duration. In the quest for high-quality RNA, we explored dehydration times of 5, 2, and 1.5 min at each ethanol gradient. Our results indicated that cells can be fully dehydrated even under these reduced durations. To further reduce the experiment time, we minimized the dehydration time to 1 min per ethanol gradient concentration. Despite this shortened duration, the cells were sufficiently dehydrated to maintain distinct morphology, enabling precise identification of target regions. As a result, we were able to omit the clearing step altogether (Fig. 1g). Our streamlined method yielded high-quality RNA, with RNA RIN values reaching up to 6.4, demonstrating suitability for downstream experiments (Fig. 3i). In conclusion, we found that a dehydration time of 1 min for each ethanol gradient concentration, coupled with the omission of clearing steps, delivers high-quality RNA most efficiently.

### Proposing a set of LMD parameters to facilitate rapid and high-quality RNA extraction

**Fig. 4:**
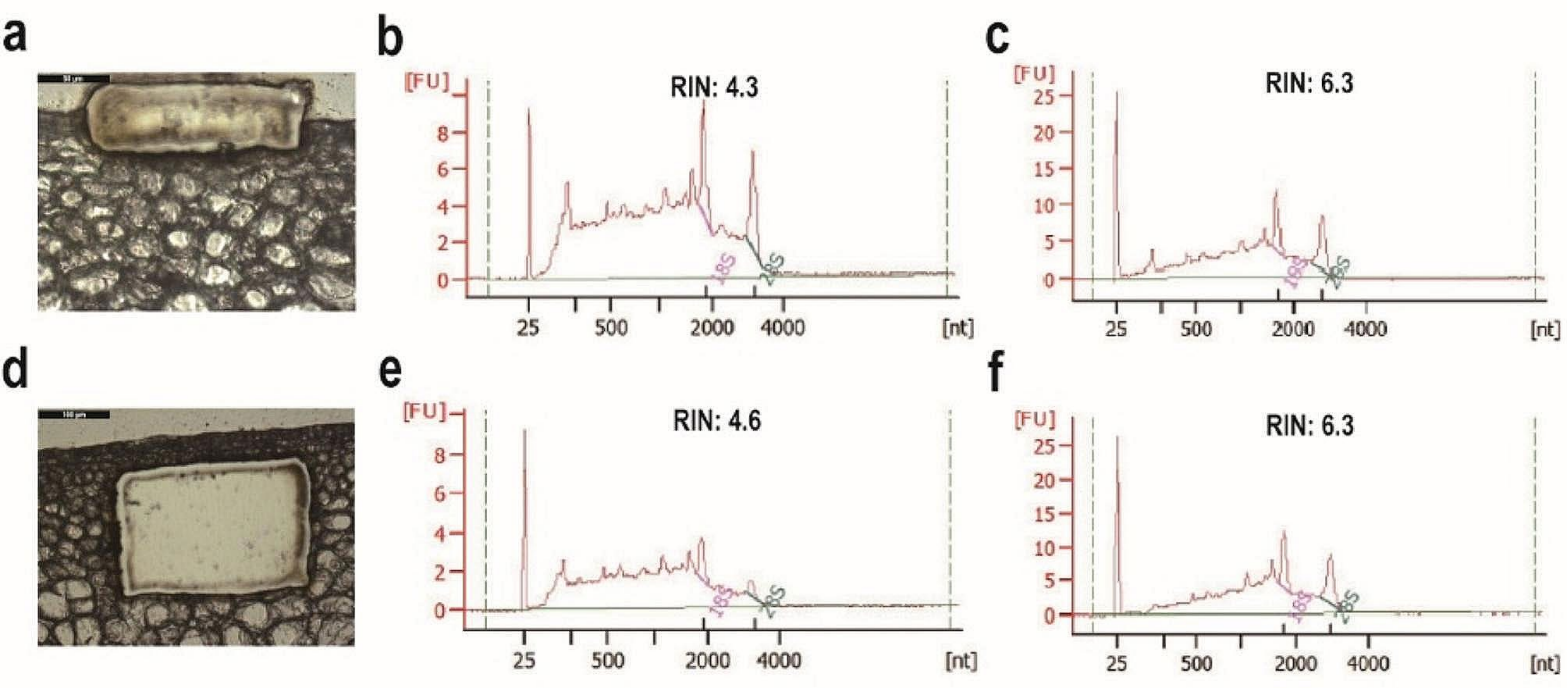
Optimization of LMD parameters for efficient RNA collection. (**a**) Cutting of epidermal cells prior to optimization. (**b**) RNA quality of epidermal cells before optimization. (**c**) RNA quality of epidermal cells after optimization. (**d**) Cutting of subepidermal cells prior to optimization. (**e**) RNA quality of subepidermal cells before optimization. (**f**) RNA quality of subepidermal cells after optimization

The LMD process is exposed to constant ribonuclease (RNase) in an open environment, which demands swift cutting of tissues to mitigate RNA degradation. An increase in the energy, aperture, speed, and frequency of the laser beam can expedite the cutting process. However, this approach can often lead to some cellular damage and compromise RNA quality (Table 1; Fig. 4a, b, d, e). Striking a balance between speed, yield, and quality is key when determining LMD parameters. Through repeated adjustments and tests, we proposed a referential set of parameters (Table 1). The fundamental principle of these parameters is to collect sufficient tissue in the shortest amount of time using settings that minimize cell damage and maximize RNA integrity. In our study, these proposed parameters facilitated the collection of ample epidermal and subepidermal cells from the citrus rind (Fig. 1h, i). We were able to complete the RNA extraction from these cells within 9 h, indicating the efficiency of our approach. These parameters not only expedited the collection process but also ensured the extraction of high-quality RNA suitable for downstream experiments (Fig. 4c, f). In summary, the set of LMD parameters we proposed can serve as a valuable reference for achieving rapid and high-quality RNA collection.

**Table 1:** Parameters of LMD for epidermal and subepidermal layers of citrus rind

Conditions	Cell layer	Magnification	Laser power	Aperture	Speed	Head current (%)	Pulse frequency
unoptimized	Epidermis	40×	60	10	8	100	4564
	Subepidermis	20×	58	10	6	100	4564
optimized	Epidermis	40×	52	3	7	100	1850
	Subepidermis	20×	46	3	6	100	1850

### High-quality transcriptomics data validate the effectiveness of our isolation method

**Fig. 5:**
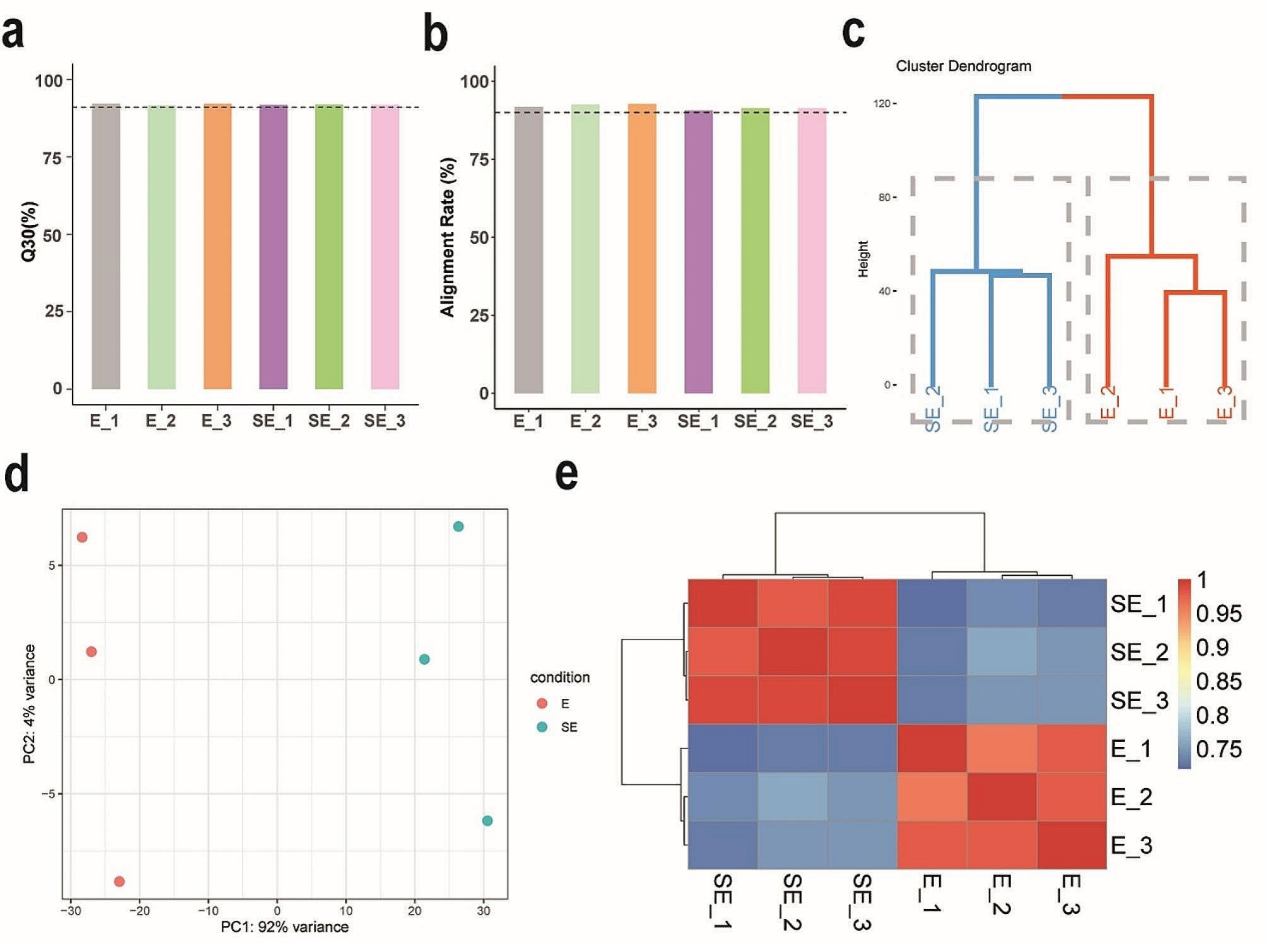
Transcriptomic analysis of citrus rind cells isolated via our LMD method. The labels ‘E’ and ‘SE’ correspond to the epidermal and subepidermal cell layers, respectively. Three biological replicates are labeled as 1, 2, and 3 for each layer. (**a**) Evaluation of Q30 average quality scores. The black dashed line represents the 91% quality cut-off on the vertical axis. (**b**) Proportion of sequenced reads aligned to the reference genome. The black dashed line indicates the 91% alignment threshold on the vertical axis. (**c**) Hierarchical clustering analysis segregating the two cell layers. Varied colors represent distinct clusters. (**d**) PCA highlighting the differential gene expression between the two cell layers. (**e**) Heatmap illustrating the correlation among samples from both cell layers, with the color gradient indicating the correlation intensity, ranging from high (red) to low (blue)

Our method proved successful in isolating pure populations of epidermal and subepidermal cells from citrus rind, which were then subjected to transcriptome sequencing. The resulting data were of good quality, with both the average Q30 quality scores and the percentage of sequenced reads mapped to the reference genome surpassing 91% (Fig. 5a, b, Supplementary Fig. 1, Additional File 1). We conducted a hierarchical clustering analysis to evaluate transcriptomic congruencies between the two distinct cell layers of the citrus rind (Fig. 5c). The formation of separate clusters for each layer validated a clear divergence in their transcriptional profiles, highlighting unique gene expression patterns and biological processes in each. To further substantiate these distinct expression patterns, we employed principal component analysis (PCA) (Fig. 5d). The PCA plot displayed a vivid separation between the samples from each layer of the rind along the first principal component, reinforcing the differential gene expression patterns unveiled in our initial clustering analysis. Moreover, a heatmap was generated to visualize the correlation matrix among various samples from the epidermal and subepidermal cell layers (Fig. 5e). The samples within each layer clustered together, suggesting a high degree of correlation among samples from the same layer. Conversely, a comparison of samples across the two layers demonstrated a low correlation between epidermal and subepidermal cells. Subsequently, we conducted differential expression analysis and identified 2,210 differentially expressed genes between epidermal and subepidermal cells. We generated a clustering heatmap showing distinct expression patterns in the two layers (Supplementary Fig. 2). GO functional enrichment analysis revealed that epidermal cells are mainly associated with wax and cutin synthesis, flavonoid biosynthesis, secondary metabolism, and defense responses (Supplementary Fig. 3). In contrast, subepidermal cells are involved in isoprenoid metabolism, cell wall thickening, and the transmembrane transport of protons, inorganic ions, and small molecules (Supplementary Fig. 4). In conclusion, our high-quality transcriptomic data not only validated the effectiveness of our isolation method but also verified its reliability in obtaining pure populations of epidermal and subepidermal cells. This robust methodological approach lays a solid groundwork for future investigations into the unique characteristics and functions of these cell layers.

## Discussion

The integration of LMD and RNA-Seq offers a powerful tool for examining specific gene expression in plants, fostering a deeper understanding of the principal genes orchestrating plant development and physiological processes [12–15]. In our research, we employed LMD to isolate cells from citrus rind and devised a more efficient, streamlined LMD protocol (Supplementary Fig. 5). The high-quality transcriptomic data acquired revealed the heterogeneity of cells within the citrus rind. This research provides a beneficial reference for the application of LMD in other plant tissues, thereby advancing the progress of precision cellomics in plants.

Pretreatment with fixatives such as formalin-acetic acid-alcohol or paraformaldehyde, in conjunction with cryoprotectants, can mitigate damage induced by ice crystal formation during cryosectioning [25–27]. A mixture of 75% ethanol and 25% acetic acid has demonstrated efficacy in the pretreatment of corn kernels and tomato fruit sections. Applying subsequent treatments of 10%, 15%, or 20% sucrose has been shown to preserve cell morphology, making this approach suitable for LMD [11, 26]. However, when extended to Arabidopsis root samples, outcomes varied. Sucrose solutions at 10% and 15% were found to maintain cellular morphology, albeit at the expense of RNA quality, while a 34% sucrose solution enhanced RNA quality but compromised cellular morphology [25]. These discrepancies underscore the necessity of tailoring fixatives and cryoprotectants to specific plant species and tissue types. In contrast, the citrus rind, endowed with sturdy cells and a stable structure, can preserve its original form during sectioning, thereby curtailing the risk of cell damage. Moreover, its lower water content diminishes the likelihood of ice crystal formation. Therefore, pretreatment is not necessary for cryosectioning citrus rinds, which also reduces the risk of RNA cross-contamination among neighbouring cells [26, 28, 29]. Direct embedding of fruit and leaf samples in OCT medium, using Peel-A-Way disposable plastic tissue embedding moulds (Polysciences Inc., Warrington, PA, USA), followed by rapid freezing, has been shown to preserve cell morphology and yield RNA of a quality level suitable for further analysis [18, 23, 30]. Nevertheless, the mould’s size can complicate the positioning of smaller samples and necessitates a significant volume of OCT compound. In our study, we innovatively embedded citrus rind in OCT medium within the cap of a 1.5 ml centrifuge tube, which was then rapidly frozen using liquid nitrogen. Not only was this method efficient and quick, but it also economized the usage of OCT medium (Fig. 1). However, our method can be referenced and slightly modified to accommodate different species, tissue types, and sample sizes.

Section thickness is a key determinant in LMD outcomes. Excessively thick sections can obscure cellular morphology and can lead to cross-contamination between distinct cells. As the thickness of a section increases, it necessitates a longer dehydration period, which concomitantly increases the risk of RNA degradation. Additionally, thicker sections can hinder the efficiency of laser penetration and cutting. On the other hand, sections that are too thin can experience cell breakage and substantial RNA degradation [31]. Furthermore, thin sections provide less cytoplasm per cut, requiring extended cutting times to gather sufficient RNA. This prolonged exposure to air escalates the risk of RNA degradation. Research on radish (*Raphanus sativus*) cortical parenchyma section thickness suggests that 10 μm is optimal for mature tissues, while 6 μm is more suitable for smaller, developing tissues. Notably, a 10 μm section thickness has yielded successful RNA extraction from tomato and citrus rind Sects. [17, 23, 32]. However, our study revealed substantial cell fragmentation and RNA degradation at 10 μm in the citrus rind, potentially due to our 170 days after full bloom (170 DAF) sampling timeline, which resulted in larger cell diameters compared to those in tomato and young citrus fruits. While Patrick et al. successfully obtained RNA suitable for RNA-Seq analysis from 20 μm Sect. [18], our study found that such sections, though preserving citrus rind structure, hindered laser cutting, complicating target tissue isolation. Upon investigating various thicknesses (8, 10, 12, 14, and 18 μm), we determined that 18 μm sections optimally preserved cellular morphology and yielded the highest quality RNA. This outcome may be attributed to the diameter of our citrus cells, which ranged from approximately 7 to 18 μm. This supports Fang et al.’s findings that thicker sections may improve RNA extraction quality [31]. In conclusion, section thickness should ideally match cell diameter to preserve cellular integrity and maximize RNA quality. Thus, our findings emphasize the importance of section thickness tailoring according to the specific characteristics of the tissue in this study.

Although post-cryosectioning, dehydration, and clearing processes are typically essential, they come with the risk of adversely affecting cellular and RNA integrity. However, previous dehydration and clearing processes were time-consuming and involved complex conditions. For instance, Matas et al. treated citrus rind cryosections with 70% ethanol at -20 °C for 15 min with three 15-minute xylene clearing cycles [23], while Collins et al. used a different regimen for apple fruit Sect. [18]. Martin et al. indicated that tomato fruit requires at least a 1-minute treatment with 70% ethanol at -20 °C, followed by 4–5 treatments with 100% ethanol [26]. In our study, however, we utilized a gradient dehydration method at room temperature with 70%, 80%, 90%, 100%, and 100% ethanol. A 1-minute dehydration interval for each concentration yielded satisfactory cell morphology suitable for LMD experiments without the necessity of a clearing process. This may be due to the thick layer of cuticle covering the epidermis of the citrus rind, which is primarily composed of wax and cutin. This layer helps the fruit reduce water content, enabling it to dehydrate rapidly. Its sturdy cellular structure negates the necessity for low-temperature procedures. Clear morphological features such as oil glands and epidermal cells can be readily discerned without a clearing step. While our approach was designed with citrus rind in mind, it may also be applicable or serve as a reference for other tissues with similar characteristics, like low water content, robust cell structure, and distinctive cellular features. For RNA samples that are sensitive or prone to degradation, or in cases where target cells are challenging to distinguish, appropriate modifications based on our method, such as incorporating low-temperature treatments or a clearing process, may be necessary. The tailoring of the protocol should, therefore, be guided by the specific properties of the tissue under investigation.

A crucial aspect of our research was investigating the optimal parameters for LMD. Fine-tuning these parameters can significantly enhance the quality of RNA and the efficiency of its collection. While previous research in this area is limited, our study underscores the importance of tailoring parameter combinations to fit the unique characteristics of different plant tissues. We discovered that parameters such as magnification, laser power, aperture, speed, head current, and pulse frequency substantially influence the quality of the collected RNA (Table 1; Fig. 4). The choice of suitable magnification hinges on the size and hardness of the target tissue cells. Higher magnification, while allowing for more precise dissection, may slow the process, reducing overall efficiency. Conversely, lower magnification can compromise dissection precision, complicating the isolation of target tissues. We found that the smaller, denser, and harder epidermal cells proved more challenging to dissect, warranting a 40× magnification for LMD. In contrast, the larger, less densely arranged subepidermal cells were more effectively dissected at a 20× magnification. Our study also highlighted the significant influence of laser power on RNA quality. Overpowering can lead to cellular damage and substantial RNA degradation, while insufficient power can impede the laser’s ability to penetrate cells, complicating the cutting process (Fig. 4). Epidermal cells, which are particularly challenging to dissect, require higher laser power. Employing a smaller laser aperture can help mitigate cellular damage and thereby preserve RNA integrity. The emission intensity should be kept below the maximum value to avoid inadvertent damage. Adjusting the pulse frequency enables control over the energy depth of each pulse. Higher frequencies result in shallower cuts, reducing thermal damage and improving cutting speed. Conversely, lower frequencies lead to deeper cuts, potentially causing thermal accumulation and sample damage [29, 30]. In summary, the LMD parameters are intertwined and mutually influential. By minimizing the laser power, reducing the aperture size, decreasing the cutting speed, and adjusting the pulse frequency, we propose a set of parameters for efficient, high-quality LMD of citrus rind (Table 1). Despite potential variations in cell size, hardness, arrangement, and slice thickness, our optimization scheme could serve as a valuable reference for future research involving cell-specific tissue separation in plants.

Our optimized protocol facilitated the rapid and precise isolation of the epidermal and subepidermal layers of the citrus rind, yielding high-quality transcriptomics data. Hierarchical clustering, PCA analysis, and heatmaps between samples show significant differences between the two cell layers. Differential gene GO function enrichment indicates that epidermal cells are primarily associated with wax and cutin synthesis, flavonoid biosynthesis, secondary metabolism, and defense responses, consistent with Matas et al. [23]. This likely reflects their role in protecting the plant from environmental stress and disease. Additionally, they found that subepidermal cells are mainly involved in photosynthesis, energy-related processes, and cell wall biosynthesis. The difference in our findings may be due to our selection of pre-break rind, where photosynthetic activity decreases and energy demands change, whereas Matas et al. studied young, expanding rind with active photosynthesis and cell wall biosynthesis. In conclusion, our transcriptome data allowed us to comprehensively and accurately understand the interlayer gene regulation and functional differences involving cellular function, metabolic pathways, signaling, and more. While our protocol is designed for citrus rind, its potential application extends to other tissues with similar characteristics, making it a valuable reference for future cell-specific LMD studies.

Single-cell research represents a dynamic and evolving field [1–3]. The combination of LMD with RNA-Seq, unlike whole tissues/organs studies, offers single-cell resolution. This enables a detailed analysis of gene regulation during development and the identification of key genes and pathways. Our optimized LMD protocol allows swift acquisition of targeted tissues for downstream experiments, thus expanding the horizons of single-cell research.

## Conclusions

We have devised a simple and rapid protocol for cell-specific transcriptome analysis via LMD, with our insights derived from citrus rind studies. This innovative method not only offers broad potential applicability but is also the most user-friendly approach reported thus far. It can serve as a valuable reference for studies on other plant tissues, thereby enriching the accuracy of histological studies at the cellular level and having broader implications for the field.

## Materials and methods

### Sample preparation

Citrus fruits from the ‘Zong Cheng’ (*C. sinensis L. Osbeck*) variety were harvested at 170 DAF. The surface of each fruit was cleaned by wiping it with a clean paper. Subsequently, the rind was carefully dissected into pieces of 8 mm x 1 mm x 1 mm using a sharp blade. These pieces were promptly placed in the cap of a 1.5 ml centrifuge tube filled with OCT medium, ensuring correct positioning of the rind with the aid of forceps. The tube, with its cap facing downwards, was then quickly frozen by immersion in liquid nitrogen for approximately 30 s. The freezing process continued for approximately another minute in liquid nitrogen, with the tube oriented in any direction. Following this, the samples were either prepared for immediate cryosectioning or stored at -80 °C.

### Cryosectioning

Cryosectioning was carried out using a Leica CM1950 cryostat. All tools that could come into contact with the samples, including brushes, sticky pens, and blades, were thoroughly cleaned with RNase Zap (Invitrogen™ AM9782, USA) to eliminate any RNase. Both the cryostat and the freezing probe were precooled for 30 min to -22 to -25 °C and − 21 to -24 °C, respectively, with concurrent UV light sterilization for 30 min. After sterilization, the embedded samples were placed in the cryostat and allowed to acclimate to the temperature for 3 min before cryosectioning began. Initial sections were cut to a thickness of 30–45 μm, with the thickness adjusted to 8, 10, 12, 14, and 18 μm for subsequent sections. The samples were adhered to the PEN membrane slide by leveraging the temperature difference between the slide and the samples, with approximately 15–20 samples prepared this way. The sections were then quickly moved to a presterilized, ultraclean workbench where they were sequentially dehydrated in 70%, 80%, 90%, and two rounds of 100% ethanol (prepared with DEPC water) for 1 min each. After a 2-minute air-drying period on the workbench, the sections were immediately subjected to laser LMD.

### LMD and RNA extraction

A Leica LMD7000 instrument was utilized for LMD prior to use, the workspace was sterilized using a 75% ethanol spray, and the bench along with all tools were cleaned with RNase Zap. To safeguard the dissected cells from RNase degradation, they were collected in an RNase-free tube containing 40 µl of RNA buffer. Optimization parameters were employed for the cuts, and each tube was processed for no more than 105 min to prevent the RNA buffer from crystallizing, which could affect subsequent RNA extraction. The collected samples were promptly stored at -80 ℃ until three tubes were filled. Total RNA extraction was executed using the Thermo Fisher Scientific Arcturus™ PicoPure™ RNA Isolation Kit (KIT0204, USA), adhering to the manufacturer’s instructions. To eliminate DNA contamination, an RNase-Free DNase Set (QIAGEN 79,254, Germany) was utilized. The quality and integrity of the extracted RNA were assessed using the Agilent RNA 6000 Pico Kit (Agilent Technologies 5067 − 1513, USA) and the Agilent 2100 Bioanalyzer (Agilent Technologies, USA).

### Library preparation and sequencing

The quality checks for the RNA from LMD samples, as well as micro RNA amplification, library construction, and sequencing, were outsourced to Annoroad Gene Technology Co., LTD (Beijing, China). The cDNA library was constructed using the Smart-Seq2 single-cell transcriptomics sequencing strategy. This process resulted in approximately 1–2 kb of amplified cDNA products, which were then purified and recovered using Beckman Ampure XP beads. The construction of each sample’s library commenced with 40 ng of the amplified cDNA product as the starting material. Libraries that met quality standards were sequenced using the PE150 paired-end program on the Illumina NovaSeq platform (Illumina, USA).

### Analysis and mapping of transcriptome data

Three biological replicates were produced for each cell layer. The raw data were filtered using Fastp software and quality-checked with FastQC. MultiQC was used to merge all quality control reports. The cleaned reads were then aligned to the SWO.v1.0 reference genome [33]. Quantitative analysis was performed using FeatureCounts from the Subread package. The quantitative outputs were analyzed with PCA plots and cluster maps, and the heatmaps were visualized in RStudio according to the methods described previously [18, 20].

### Electronic supplementary material

Below is the link to the electronic supplementary material.

Supplementary Material 1

Supplementary Material 2

Supplementary Material 3

Supplementary Material 4

Supplementary Material 5

Supplementary Material 6

## Data Availability

The datasets used and analysed during the current study are available from the corresponding author on reasonable request.

## Abbreviations

LMD: Laser microdissection
OCT: Optimal Cutting Temperature
RNA-Seq: RNA Sequencing
RNase: Ribonuclease
