# Supplementary figures and images for: Developing a simple and rapid method for cell-specific transcriptome analysis through laser microdissection: insights from citrus rind with broader implications
Source: Plant Methods. 2024 Jul 27;20:113. doi: 10.1186/s13007-024-01242-y (PMC11282741; doi:10.1186/s13007-024-01242-y)

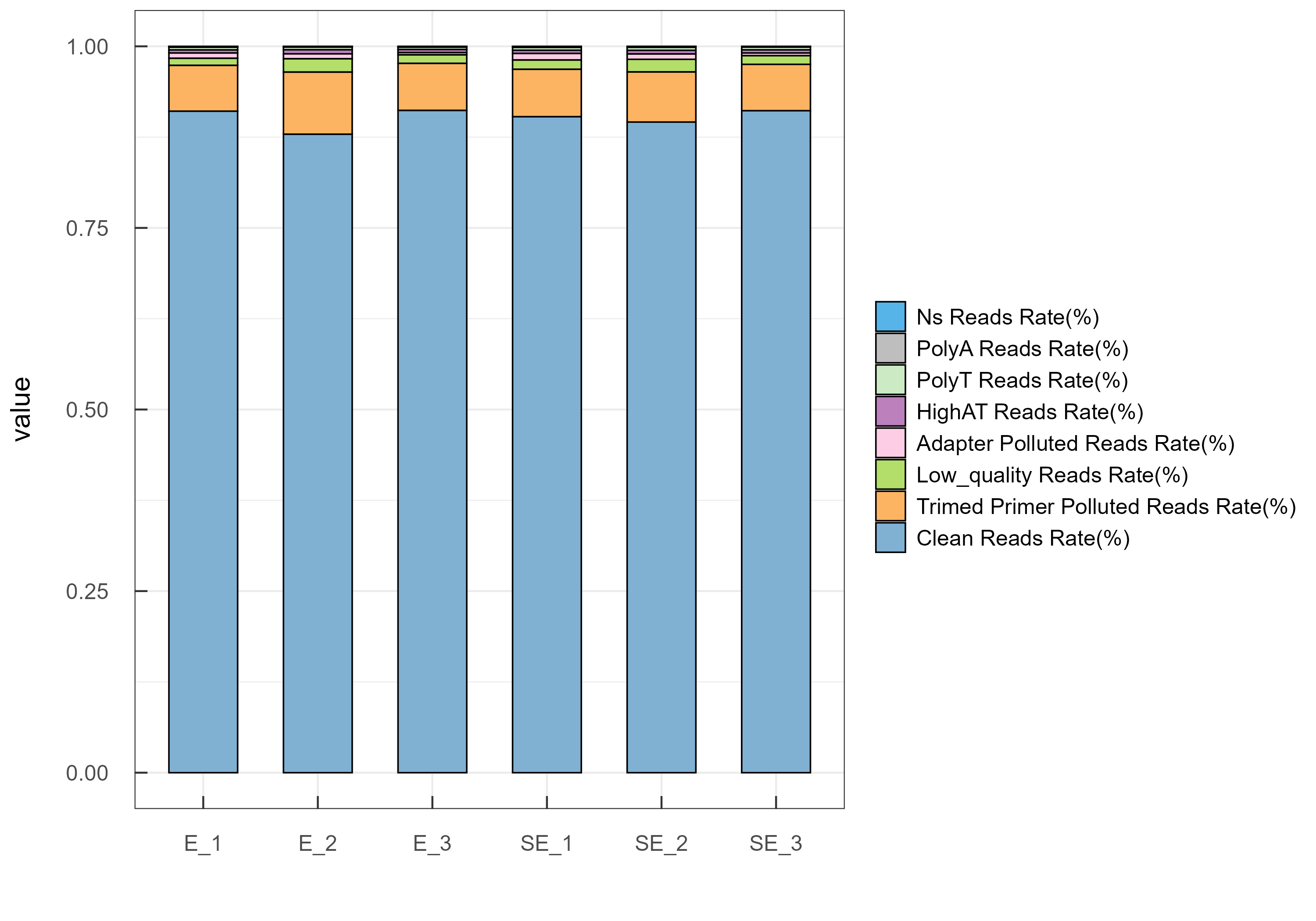

Supplement: Supplementary file 1 — Supplementary Material 1 [file 13007_2024_1242_MOESM1_ESM.png]

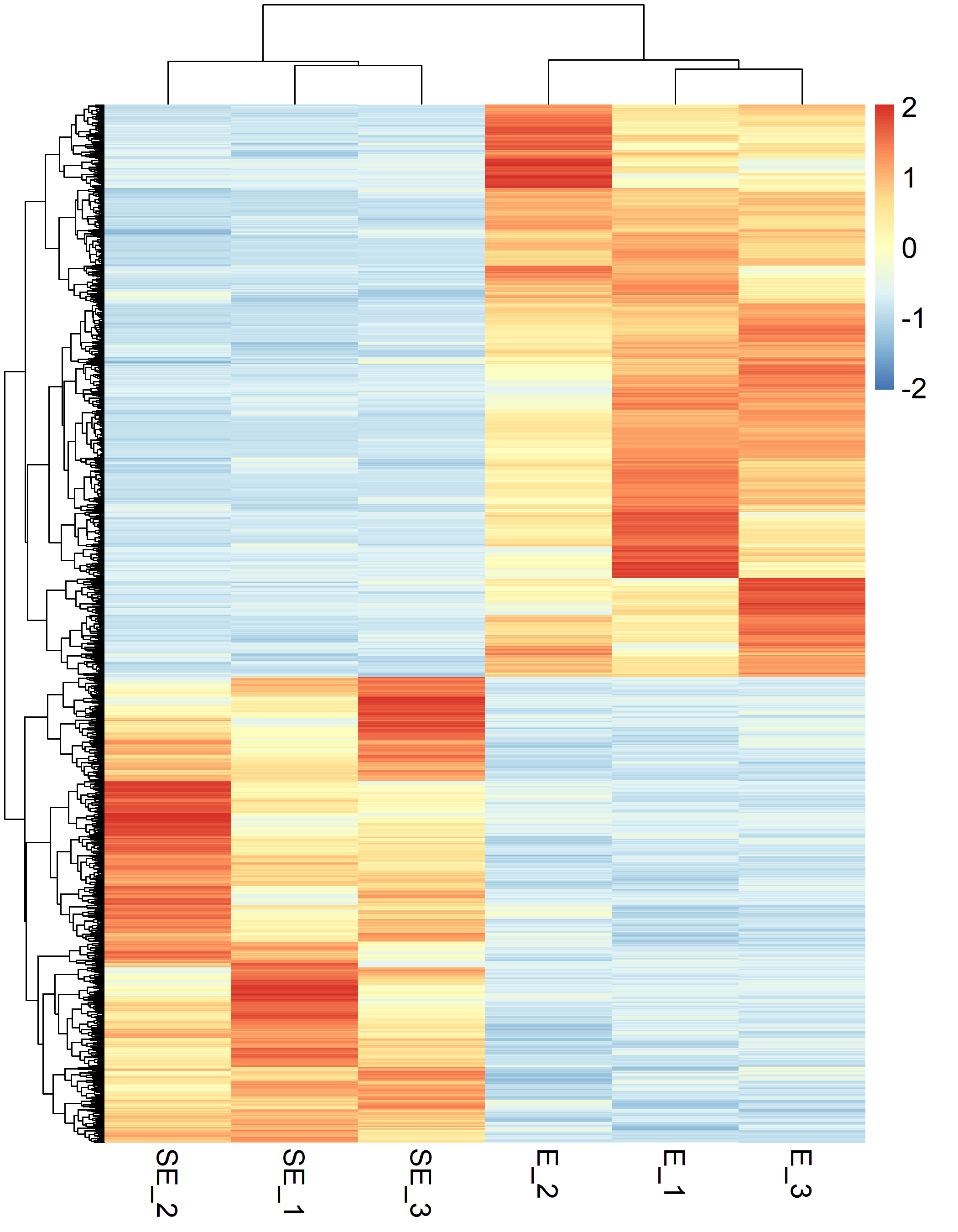

Supplement: Supplementary file 3 — Supplementary Material 3 [file 13007_2024_1242_MOESM3_ESM.png]

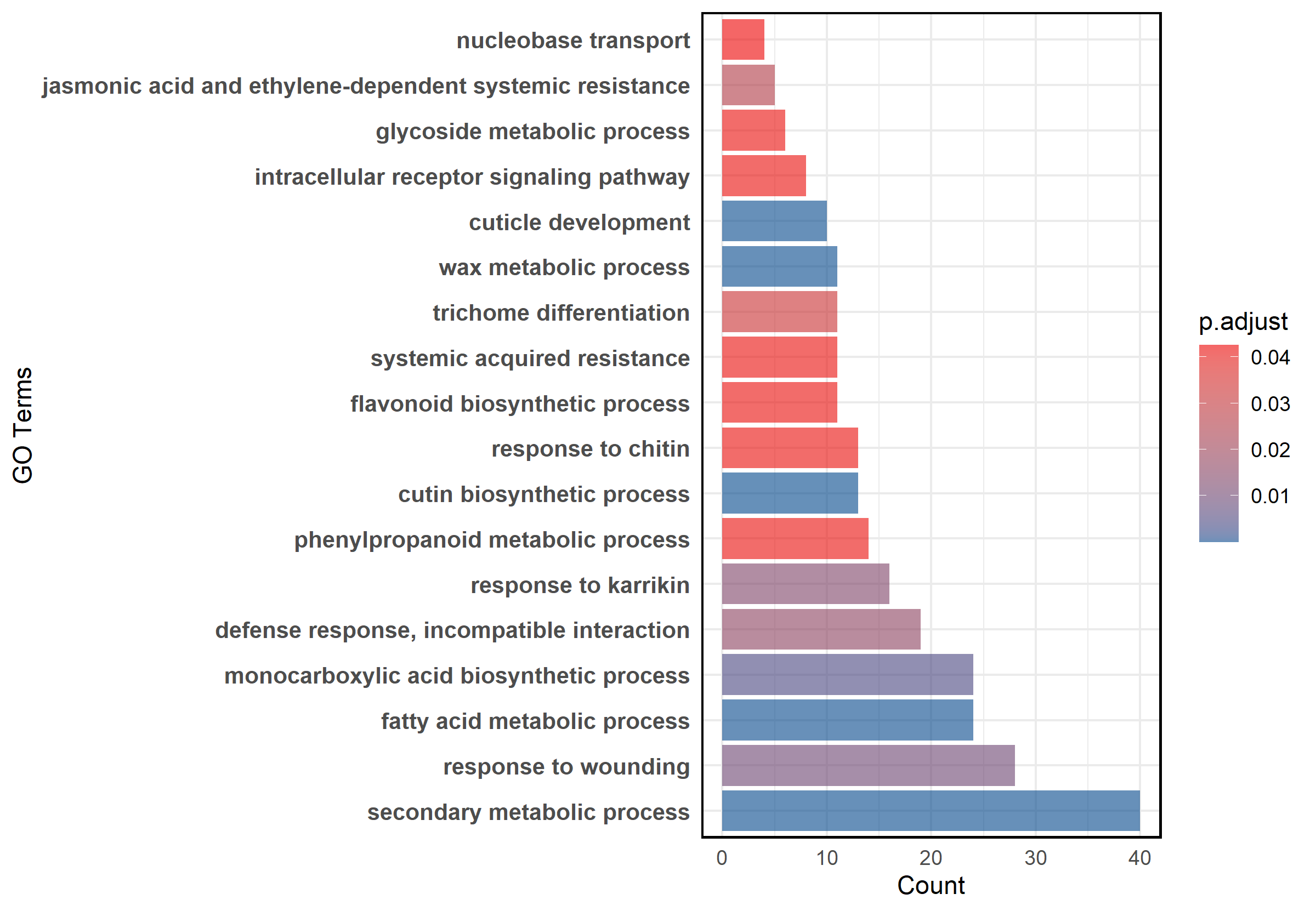

Supplement: Supplementary file 4 — Supplementary Material 4 [file 13007_2024_1242_MOESM4_ESM.png]

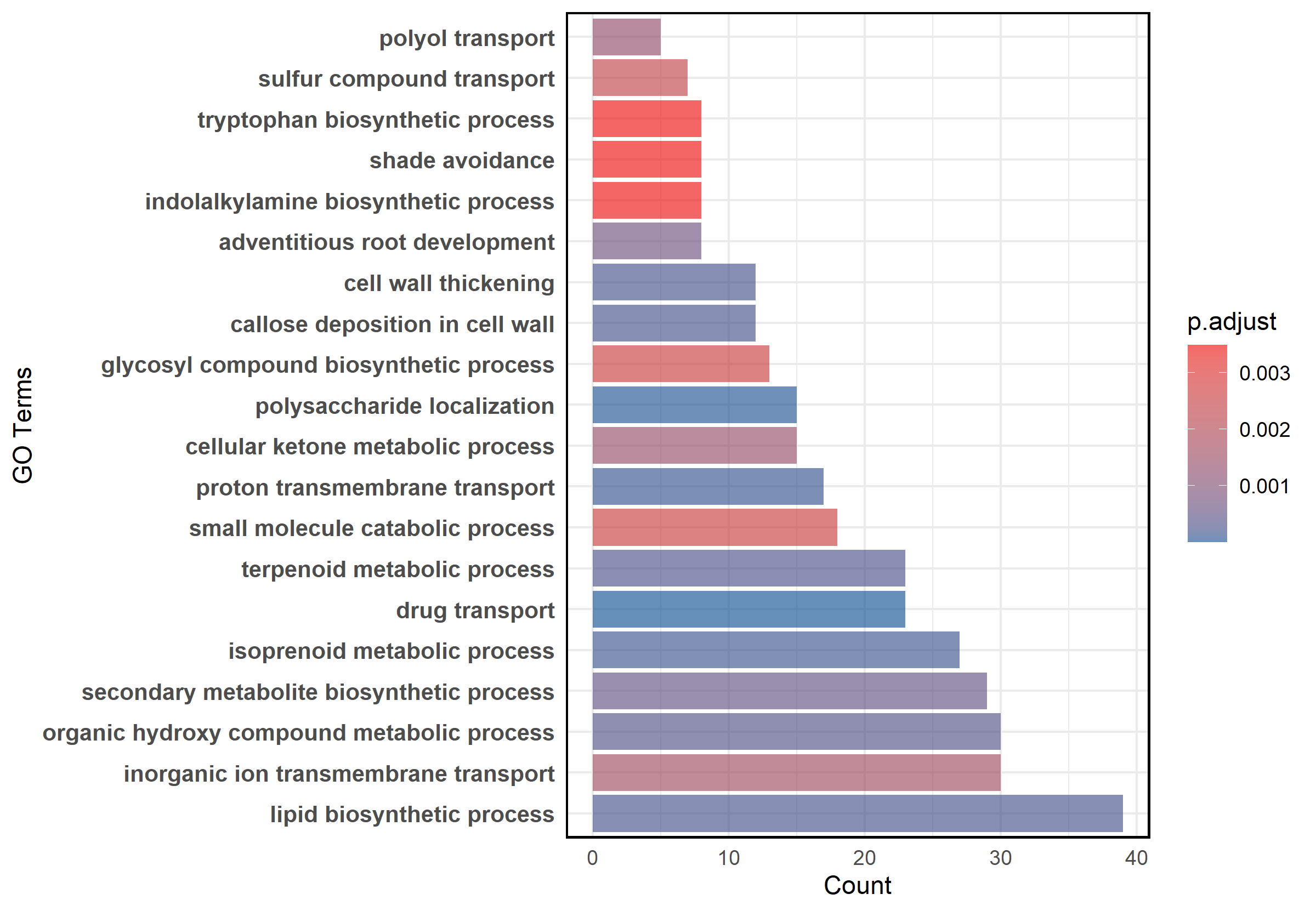

Supplement: Supplementary file 5 — Supplementary Material 5 [file 13007_2024_1242_MOESM5_ESM.png]

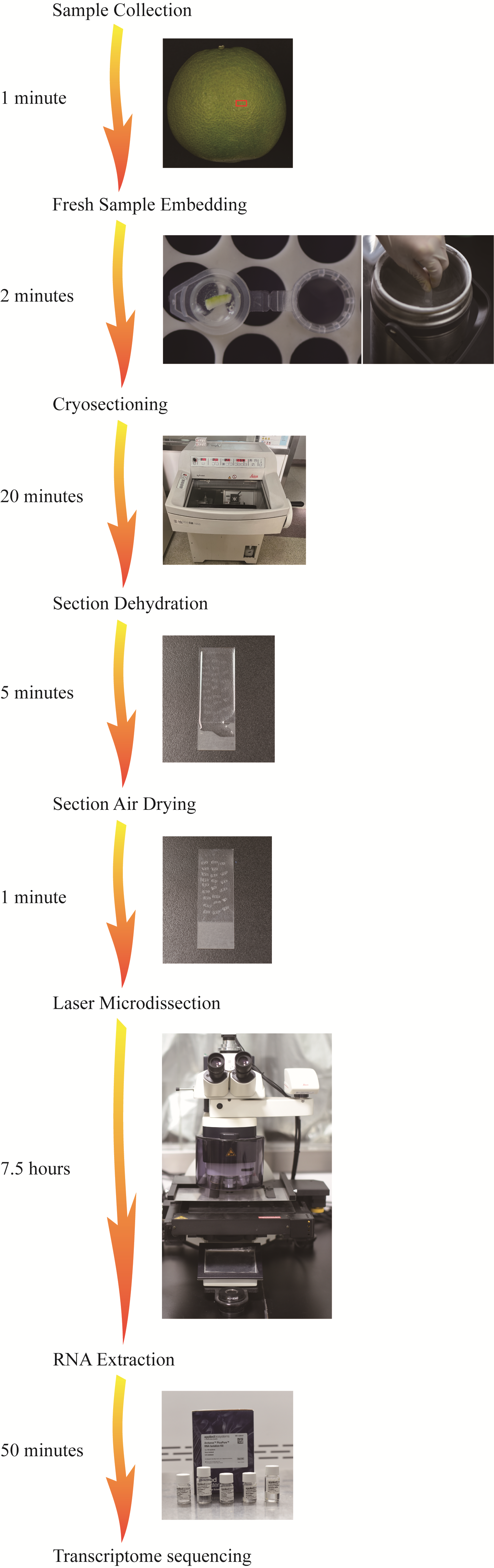

Supplement: Supplementary file 6 — Supplementary Material 6 [file 13007_2024_1242_MOESM6_ESM.png]
