# Supplementary material for: Developing a simple and rapid method for cell-specific transcriptome analysis through laser microdissection: insights from citrus rind with broader implications
Source: Plant Methods. 2024 Jul 27;20:113. doi: 10.1186/s13007-024-01242-y (PMC11282741; doi:10.1186/s13007-024-01242-y)

A modular tool to aggregate results from bioinformatics analyses across many samples into a single report.

General Statistics

Copy table | Configure Columns | Plot | Showing 12/12 rows and 3/6 columns.

Sample Name	% Dups	% GC	M Seqs
**E_1_R1**	71.5%	45%	33.3
**E_1_R2**	68.8%	45%	33.3
**E_2_R1**	77.9%	45%	34.8
**E_2_R2**	74.9%	45%	34.8
**E_3_R1**	69.7%	45%	40.8
**E_3_R2**	66.5%	45%	40.8
**SE_1_R1**	66.4%	44%	37.5
**SE_1_R2**	64.0%	44%	37.5
**SE_2_R1**	79.5%	45%	33.8
**SE_2_R2**	76.6%	45%	33.8
**SE_3_R1**	73.1%	44%	38.2
**SE_3_R2**	69.8%	44%	38.2

FastQC

FastQC is a quality control tool for high throughput sequence data, written by Simon Andrews at the Babraham Institute in Cambridge.

Sequence Counts

Sequence counts for each sample. Duplicate read counts are an estimate only.

Number of reads | Percentages

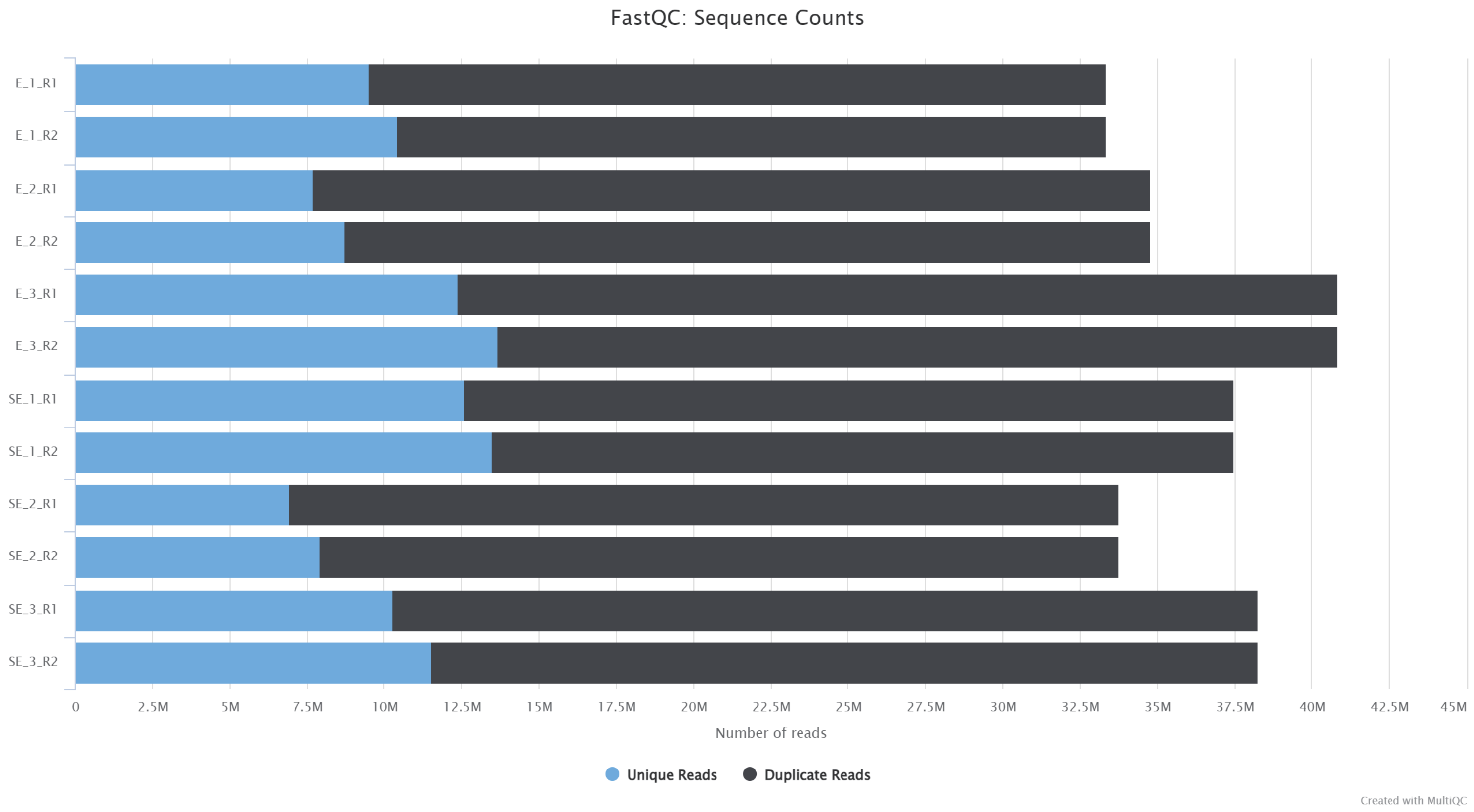

Sequence Quality Histograms

12

The mean quality value across each base position in the read.

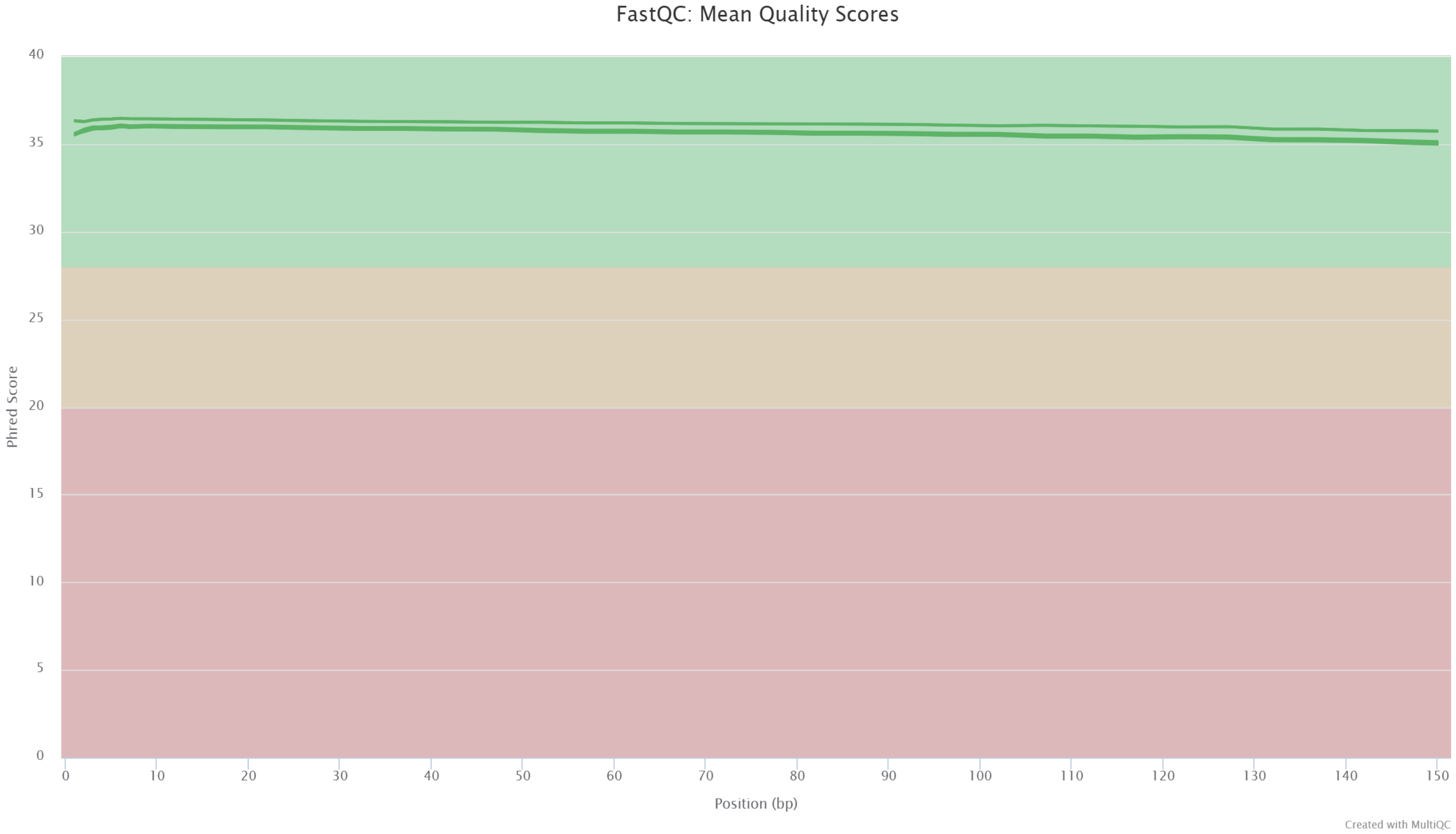

Per Sequence Quality Scores

12

The number of reads with average quality scores. Shows if a subset of reads has poor quality

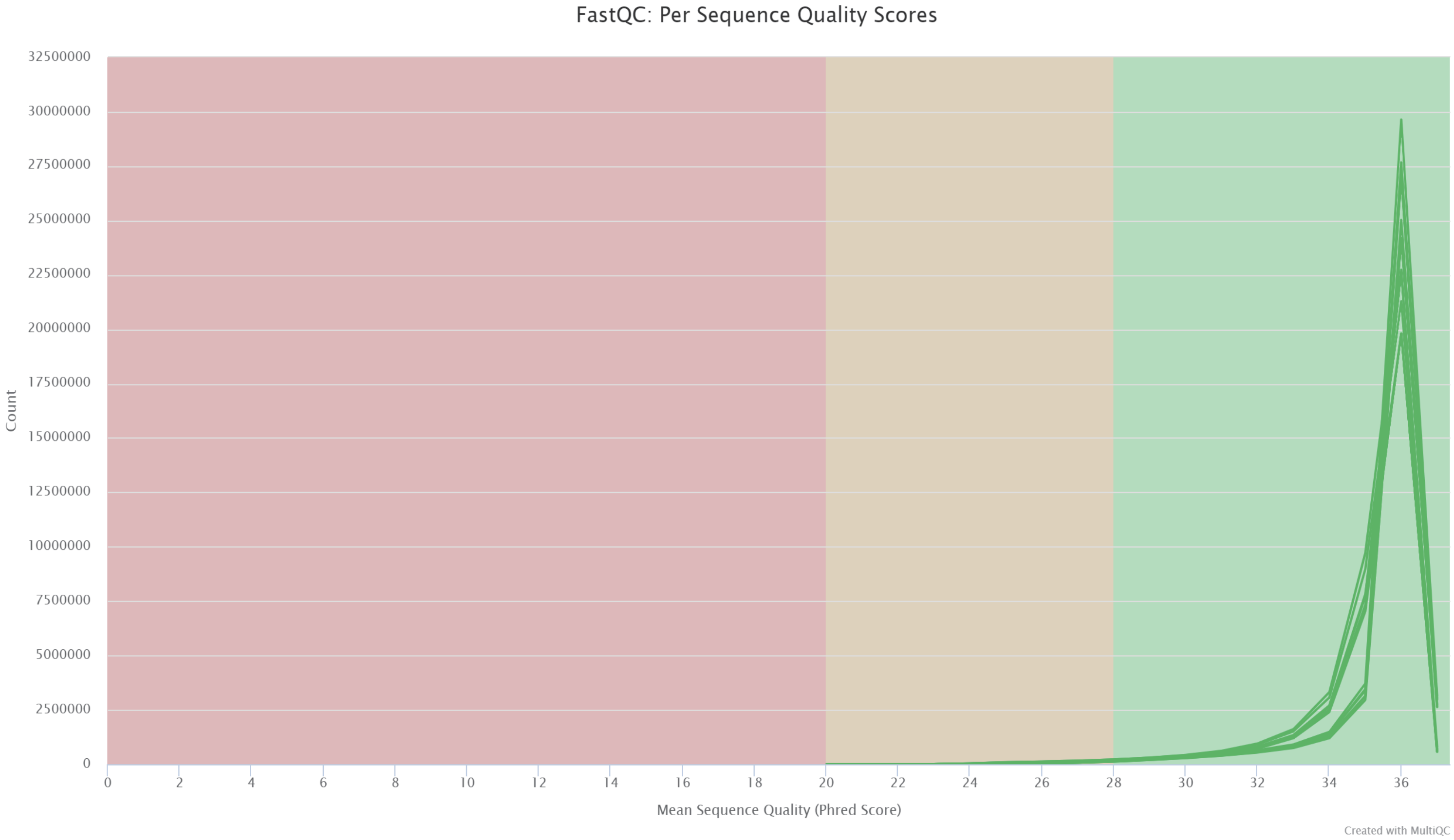

Per Base Sequence Content

10 2

The proportion of each base position for which each of the four normal DNA bases has been called.

☒ Click a sample row to see a line plot for that dataset.

☒ Rollover for sample name

Position: - %T: - %C: - %A: - %G: -

☒ Export Plot

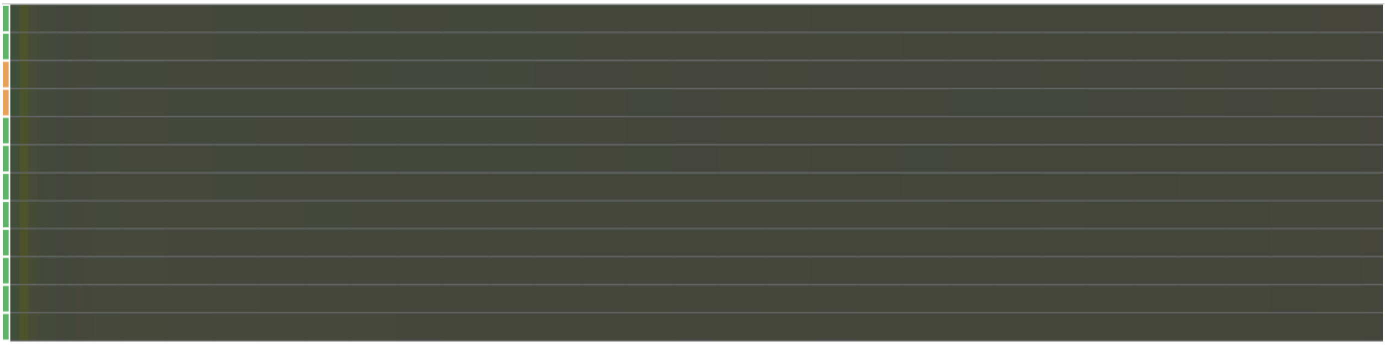

Per Sequence GC Content

12

The average GC content of reads. Normal random library typically have a roughly normal distribution of GC content.

Percentages | Counts

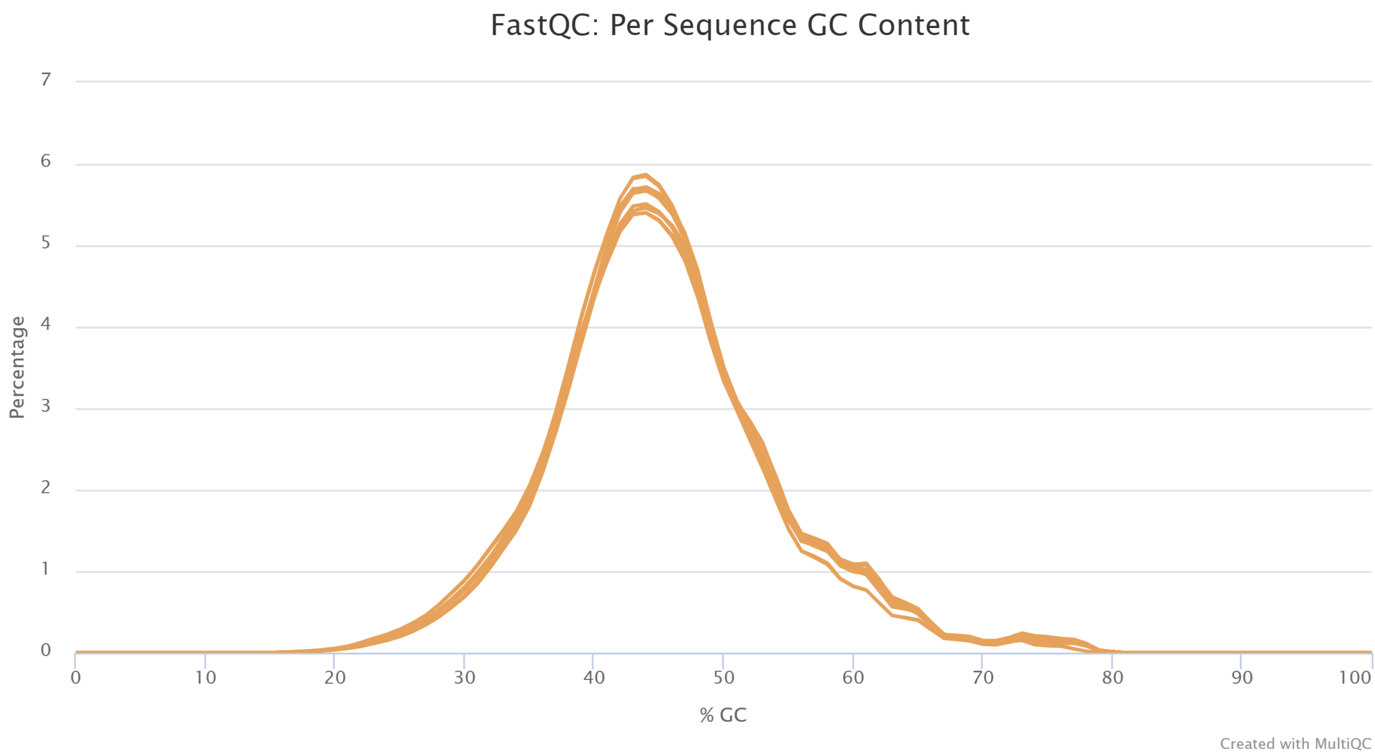

Per Base N Content

12

The percentage of base calls at each position for which an `N` was called.

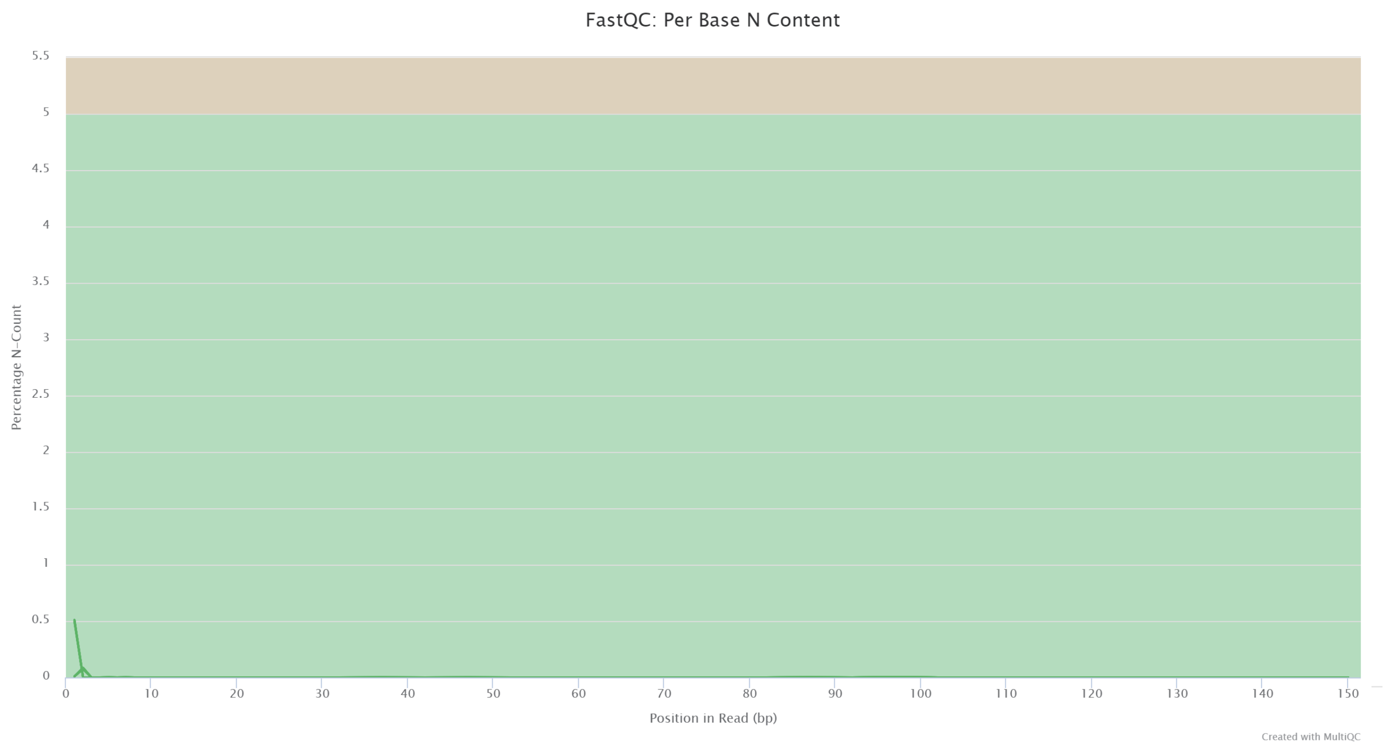

Sequence Length Distribution

12

The distribution of fragment sizes (read lengths) found. See the FastQC help

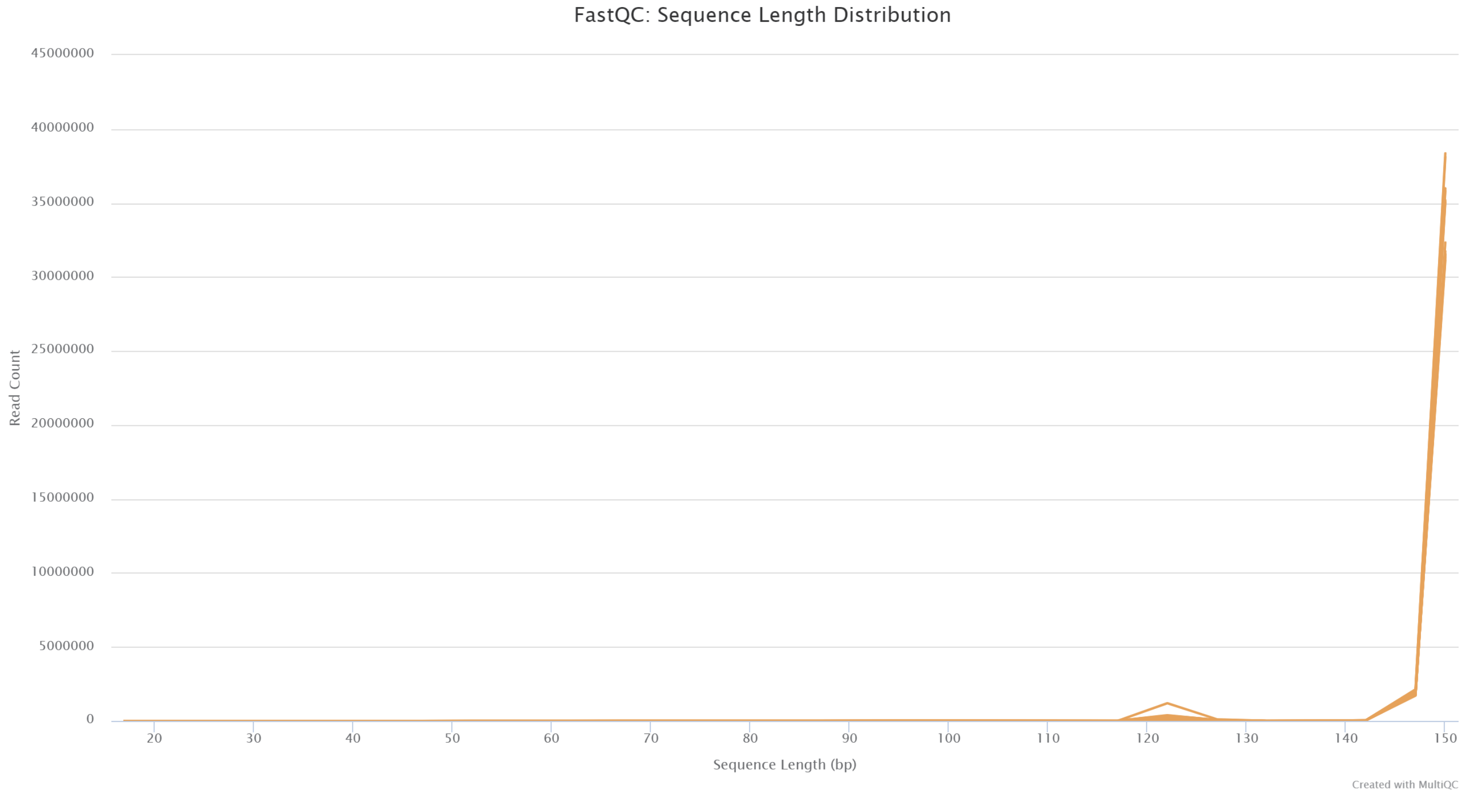

Sequence Duplication Levels

The relative level of duplication found for every sequence.

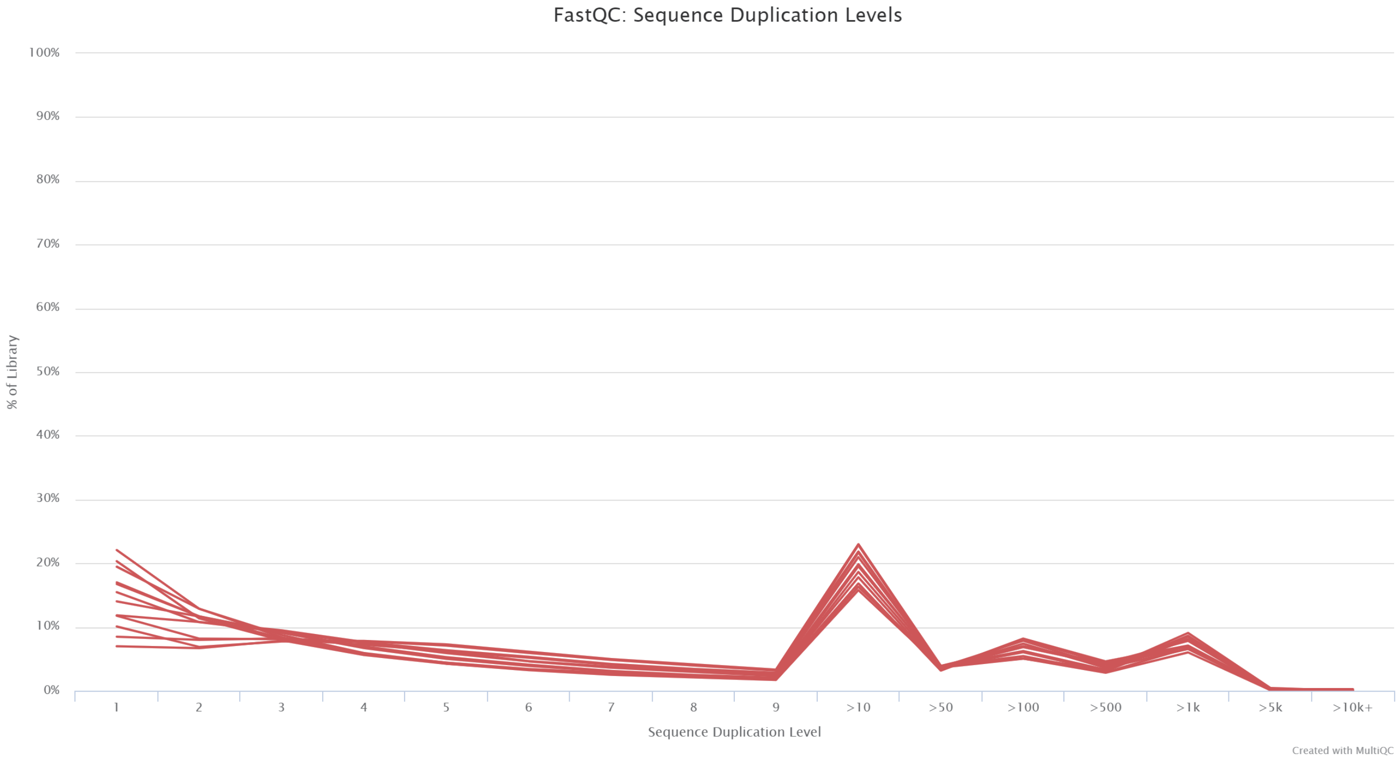

Overrepresented sequences 10 2

The total amount of overrepresented sequences found in each library.

12 samples had less than 1% of reads made up of overrepresented sequences

Adapter Content 12

The cumulative percentage count of the proportion of your library which has seen each of the adapter sequences at each position.

No samples found with any adapter contamination > 0.1%

Status Checks

Status for each FastQC section showing whether results seem entirely normal (green), slightly abnormal (orange) or very unusual (red).

Min: 0 **Max:** 1

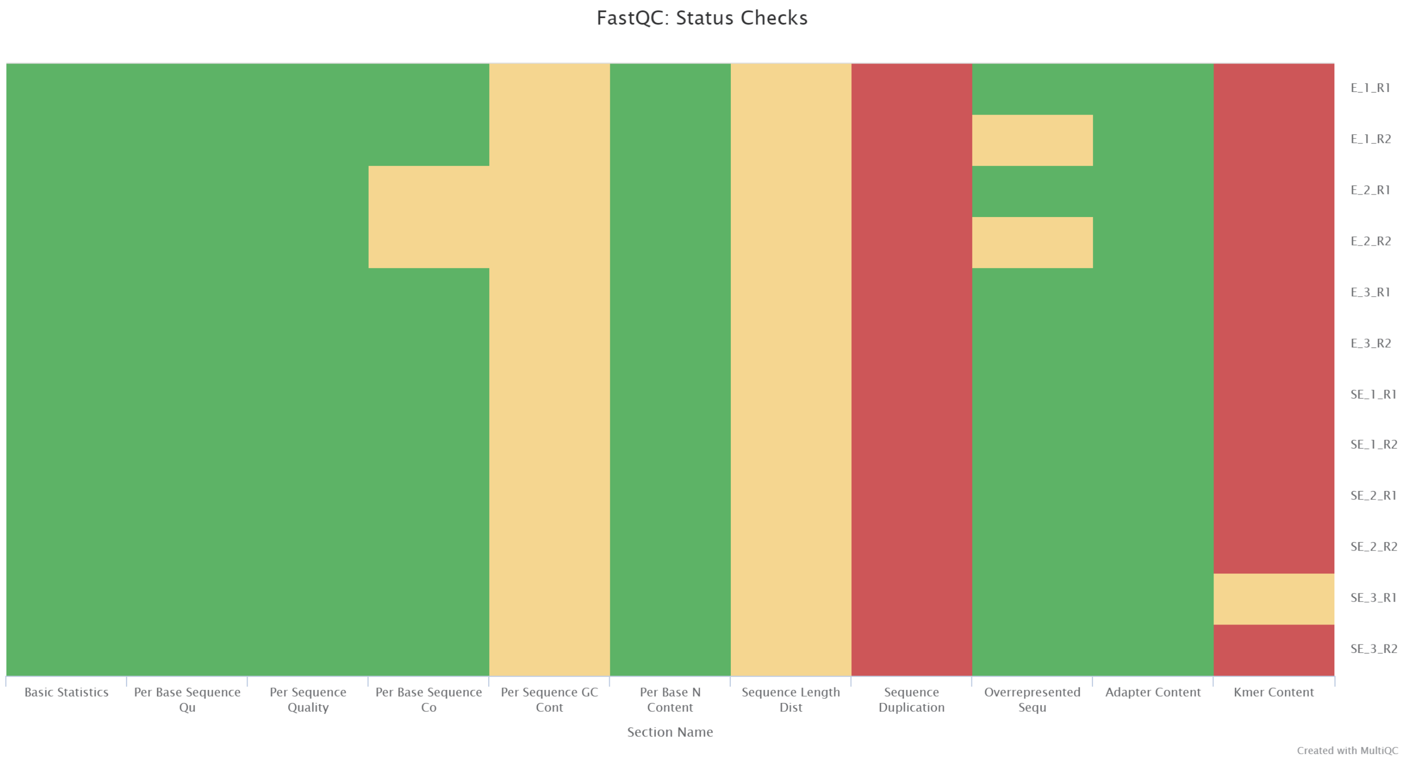

Supplement: Supplementary file 2 — Supplementary Material 2 [file 13007_2024_1242_MOESM2_ESM.pdf]
